# Hydrogen peroxide induces La cytoplasmic shuttling and increases hepatitis C virus internal ribosome entry site-dependent translation

**DOI:** 10.1099/jgv.0.000556

**Published:** 2016-09

**Authors:** Shiu-Wan Chan

**Affiliations:** ^1^​ Faculty of Biology, Medicine and Health, The University of Manchester, Manchester, UK

**Keywords:** hepatitis C virus, IRES, translation, hydrogen peroxide, oxidative stress, La

## Abstract

We have previously shown that physio/pathological levels of hydrogen peroxide (H_2_O_2_) stimulate translation from the hepatitis C virus (HCV) internal ribosome entry site (IRES) element in tissue-cultured cells. Here, using *in vitro* translation, we further show that H_2_O_2_ upregulates HCV IRES-dependent mRNA translation and correlates with an increase in intracellular oxidant level. Using Western blotting, immunocytochemistry, microscopy and affinity pulldown, we show that H_2_O_2_ stimulates HCV IRES-dependent translation and correlates with nuclear–cytoplasmic shuttling of the La autoantigen, resulting in enhanced binding of cytoplasmic La to HCV IRES RNA. The role of the La protein in H_2_O_2_-stimulated IRES-dependent translation is further confirmed by the ability of an anti-La antibody to suppress H_2_O_2_-activated IRES-dependent translation *in vitro*. This is further supported by the ability of an ectopically expressed dominant, negative La mutant protein to suppress H_2_O_2_-inducible IRES-mediated translation in Huh7 cells, transiently transfected with a bicistronic reporter and in a sub-genomic replicon cell line resembling a persistent infection. On the other hand, translation from the encephalomyocarditis virus IRES is diminished in the presence of H_2_O_2_, suggesting that H_2_O_2_ translational responsiveness is a specific property of the HCV IRES and is not a general phenomenon for all viral IRESs. Altogether, these results suggest that HCV adapts to physio/pathological oxidative stress in the host cell by mediating La cytoplasmic shuttling to enhance its IRES-dependent translation.

## Introduction

The hepatitis C virus (HCV) is a single-stranded, positive-sense RNA virus belonging to the genus *Hepacivirus* of the family *Flaviviridae* (Chan, 2014). Its 9.6 kb genome encodes a single polypeptide, which is cleaved by the host and viral proteases into structural proteins core, envelopes E1 and E2, and non-structural proteins p7, NS2, NS3, NS4A, NS4B, NS5A and NS5B. HCV poses a major threat to human health, causing chronic hepatitis, which can then progress into cirrhosis and/or hepatocellular carcinoma. There is now accumulating evidence that oxidative stress may be responsible for the pathogenesis of viral hepatitis and other forms of liver diseases (Paracha *et al.*, 2013). Oxidative stress is a prominent clinical feature associated with HCV infection. Hepatitis C patients frequently present elevated pro-oxidant and reduced anti-oxidant levels in the blood and liver, with iron overload, increased lipid peroxidation and oxidative DNA damage and decreased hepatic glutathione (Fujita *et al.*, 2008; Konishi *et al.*, 2006; Paradis *et al.*, 1997; Venturini *et al.*, 2010). Proteomic and microarray analysis of liver biopsies revealed increased oxidative stress in hepatitis C samples (Diamond *et al.*, 2007; Yamashita *et al.*, 2001). Little is known about how the virus can survive in such a highly oxidative environment. Our previous demonstration that HCV translation was upregulated by physio/pathological doses of hydrogen peroxide (H_2_O_2_) in hepatocytes suggests a translational advantage of the virus in an oxidative environment, enabling the virus to survive and establish chronicity (MacCallum *et al.*, 2006).

Translation from the HCV genome is mediated by an internal ribosome entry site (IRES) element (Tsukiyama-Kohara *et al.*, 1992). The IRES element mediates an alternative form of translation distinctive from that of cap-dependent translation of the vast majority of cellular genes, allowing selective translation of genes under conditions when global protein synthesis is compromised (Jackson, 2005). Cap-dependent translation is regulated by a canonical set of eukaryotic initiation factors (eIFs). In contrast, translation from the IRES element requires only a limited number of eIFs. In addition, IRES-dependent translation is regulated by unique sets of IRES *trans*-acting factors (ITAFs).

A number of putative ITAFs have been identified for the HCV IRES element, including the autoantigen La (Costa-Mattioli *et al.*, 2004). La is pivotal in mediating translation from a number of IRESs (Costa-Mattioli *et al.*, 2004; Holcik & Korneluk, 2000; Petz *et al.*, 2012). La plays a critical role in HCV IRES-dependent translation initiation, by binding to and altering the conformation of the IRES element to orchestrate assembly of the ribosomal complex (Pudi *et al.*, 2004).

ITAF modification is an important aspect in the regulation of IRES activity under stress conditions, using mechanisms such as nuclear–cytoplasmic shuttling, protein cleavage, phosphorylation and increased protein expression (Brenet *et al.*, 2009; Lewis & Holcik, 2008; Sella *et al.*, 1999; Shiroki *et al.*, 1999). La modification has also been implicated in playing a regulating role in IRES-dependent translation initiation under various stress conditions (Petz *et al.*, 2012; Shiroki *et al.*, 1999; Zhang *et al.*, 2012). In this study, we therefore investigated the role of La and the mechanism by which La upregulates HCV IRES-dependent translation under oxidative stress conditions.

## Results

### H_2_O_2_ induces host-factor change to stimulate IRES-dependent translation

**Fig. 1.**
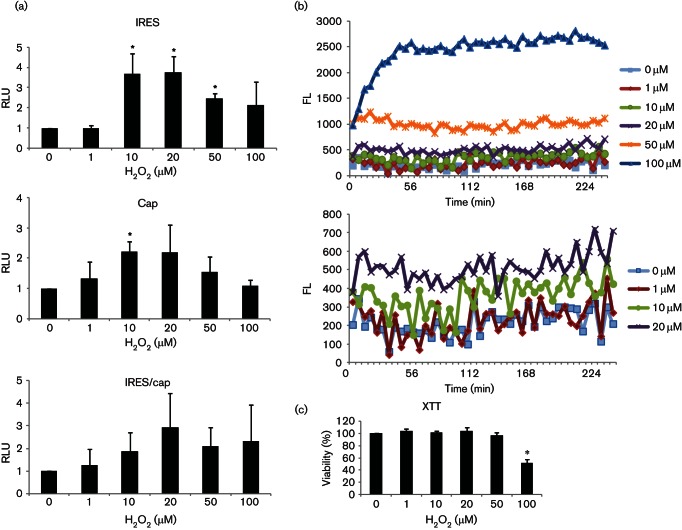
H_2_O_2_ stimulates translation *in vitro*. (a) Cytosolic S10 fractions extracted from Huh7 cells treated with doses of H_2_O_2_, as indicated, for 1 h, were used to prime *in vitro* translation programmed with the bicistronic pRL1b reporter transcript. The HCV IRES and cap-translational activities were measured by firefly and *Renilla* luciferase activities, respectively, and expressed relative to the untreated controls, which are set as 1. The IRES/cap ratio is represented by the ratio of firefly-to-*Renilla* luciferase activities and is expressed relative to the untreated control, which is set as 1. The values obtained represent the mean±sem of three independent experiments, performed in duplicates. RLU, Relative luciferase units. (b) A representation of three independent dichlorofluorescin fluorometric assays, performed in quadruplicates, showing the kinetics of reactive oxygen species (ROS) generation in Huh7 cells (19 000 per well/96-well plate) after treatment with doses of H_2_O_2_, as indicated. The bottom part of the graph is enlarged and depicted below to show ROS generation in the lower range of H_2_O_2_. FL, Fluorescence units. (c) XTT assay showing viability of Huh7 cells (19 000 per well/96-well plate) after treatment with doses of H_2_O_2_, as indicated, for 24 h. The values obtained represent the mean±sem of three independent experiments, performed in quadruplicates, and are expressed relative to the untreated control, which is set as 100 %. Significance of the difference *P<0.05.

Using Huh7 cells transiently transfected with the bicistronic reporter pRL1b mRNA in which translation of the *Renilla* luciferase and firefly luciferase is driven by cap-dependent and HCV IRES-dependent translation, respectively, we have previously shown that H_2_O_2_ stimulates translation from the HCV IRES in tissue-cultured cells (MacCallum *et al.*, 2006). This translational upregulation could be brought about by H_2_O_2_-induced change in the host factor and/or in the RNA template. In order to determine the role of host factor in H_2_O_2_-activated IRES-dependent translation, we performed an *in vitro* study using cytosolic extracts harvested from H_2_O_2_-treated Huh7 cells to prime *in vitro* translation programmed with the same bicistronic RNA template containing the HCV IRES. Huh7 cells were treated with 0 µM, 1 µM, 10 µM, 20 µM, 50 µM and 100 µM of H_2_O_2_ for 1 h. Cytoplasmic S10 fractions extracted from 10 µM, 20 µM, 50 µM and 100 µM H_2_O_2_-treated cells were able to prime and enhance *in vitro* translation from the HCV IRES over that of the untreated control, suggesting that host-factor change in response to H_2_O_2_ is responsible for H_2_O_2_-activated IRES-dependent translation (Fig. 1a). A low level of exogenous H_2_O_2_ (1 µM), which did not result in any increase in intracellular oxidants, also did not stimulate IRES-dependent translation, confirming that elevated intracellular oxidant level is essential to stimulate IRES-dependent translation (Fig. 1a, b). Furthermore, a slight elevation in intracellular oxidant level, as induced by 10 µM of H_2_O_2_, was sufficient to stimulate IRES-dependent translation. However, higher intracellular oxidant levels, as induced by 50 µM and 100 µM of H_2_O_2_, stimulated IRES-dependent translation to a lesser extent than lower intracellular oxidant levels of H_2_O_2_, as induced by 10 µM and 20 µM of H_2_O_2_, due to the cytotoxic effect of higher doses of H_2_O_2_ (Fig. 1a, c). Note that although the XTT viability test did not detect significant cell death at 50 µM of H_2_O_2_, a low degree of apoptosis was always visible at this concentration, similar to what we have observed before (MacCallum *et al.*, 2006). A concentration of 100 µM of H_2_O_2_ caused significant cell death (41–58 %), resulting in significant variation in translational activity. This might explain the insignificant increase of IRES-dependent translation in Fig. 1, but significant increase of IRES-dependent translation in Fig. 6. Despite this variation, this concentration of H_2_O_2_ consistently enhanced IRES-dependent translation. In contrast, H_2_O_2_ also stimulated cap-dependent translation, but the results were not consistent and, hence, insignificant in most cases (Figs 1 and 6). This inconsistency in cap-dependent translation gave rise to insignificant increase in the IRES/cap ratio in Fig. 1, but significant increase in the IRES/cap ratio in Fig. 6. Importantly, the increase in the IRES/cap ratio was consistent in cells treated with 10–100 µM of H_2_O_2,_ confirming differential upregulation of IRES-dependent translation by physio/pathological levels of H_2_O_2_ *in vitro*.

### H_2_O_2_ upregulates IRES-dependent translation and correlates with increased cytosolic La level

**Fig. 2.**
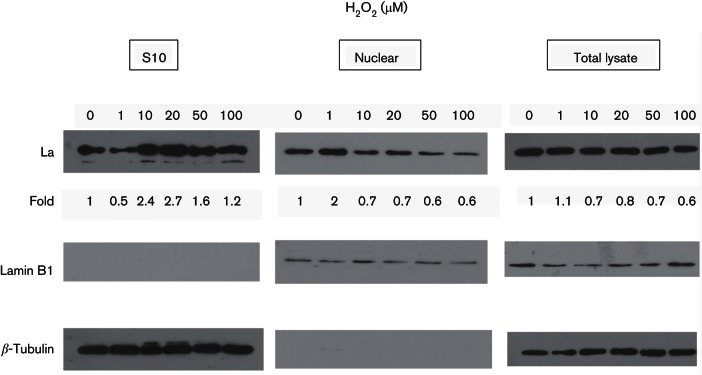
H_2_O_2_ increases the cytoplasmic La level. Western blots showing the levels of the La protein and the internal loading control *β*-tubulin/lamin B1 in the cytoplasmic S10, nuclear extracts and total lysates, in Huh7 cells treated with doses of H_2_O_2_, as indicated, for 1 h. The following concentrations were used: anti-La antibody SW5 (1 : 5000); *β*-tubulin (1 : 5000; clone TUB2.1, Sigma); lamin B1 (1 : 1000; D9V6H, Cell Signaling); HRP-conjugated anti-mouse antibody (1 : 5000; Cell Signaling); HRP-conjugated anti-rabbit antibody (1 : 500; Cell Signaling). The levels of La were quantified using ImageJ, normalized against *β*-tubulin (for S10 fractions and total lysates)/lamin B1 (for nuclear fractions) and expressed as fold increase relative to the 0 µM H_2_O_2_ control.

La is an important ITAF for many IRESs (Costa-Mattioli *et al.*, 2004; Holcik & Korneluk, 2000; Petz *et al.*, 2012). La undergoes post-translational changes in response to oxidative stress and is responsible for the oxidative stimulation of translation from the cellular IRES for Nrf2 (Zhang *et al.*, 2012). As La is a key ITAF for HCV IRES-dependent translation (Costa-Mattioli *et al.*, 2004), we therefore sought to examine whether H_2_O_2_-induced change in the La protein could account for the stimulation of HCV IRES-dependent translation. Cytoplasmic shuttling of ITAF is a rapid means of regulation of IRES-dependent translation under stress conditions (Lewis & Holcik, 2008); we therefore measured the amount of La protein in cytosolic extracts in cells exposed to H_2_O_2_ using Western blotting. Cytosolic extracts from 10 µM, 20 µM, 50 µM and 100 µM H_2_O_2_-treated cells displayed increases in the La protein level over that of the untreated control (Fig. 2). Importantly, the levels of cytoplasmic La correlated with the degrees of IRES-dependent translational stimulation by H_2_O_2_ (Figs 1a and 2). A very low level of H_2_O_2_ of 1 µM, which did not result in any increase in intracellular oxidants, also did not induce translational upregulation or led to an increase in cytoplasmic La level (Figs 1a, b and 2). Low levels of H_2_O_2_, at 10 µM and 20 µM, which resulted in the highest stimulation of IRES-dependent translational activity, also caused the accumulation of the highest level of La in the cytoplasm (Figs 1a and 2). High levels of H_2_O_2_, at 50 µM and 100 µM, which resulted in an intermediate level of stimulation of IRES-dependent translational activity, also caused the accumulation of an intermediate level of La in the cytoplasm. Examination of the nuclear fractions using lamin B1 as a marker, revealed a corresponding decrease in the La protein nuclear levels in cells treated with 10 µM, 20 µM, 50 µM and 100 µM H_2_O_2_, compared with that of the untreated cells (Fig. 2). We noticed a reduced cytoplasmic La level and an elevated nuclear La level in cells treated with 1 µM H_2_O_2_, although the significance of this is currently unknown and will need further investigation. The La protein levels from total lysates remained similar to that of the untreated control in all cases. Altogether, these results suggest that the increase in cytosolic La level could be a result of cytoplasmic shuttling. The absence of lamin B1 – a nuclear marker – in the S10 fractions, and *β*-tubulin – a cytoplasmic marker – in the nuclear fractions, confirms the purity of the fractionation process. The absence of the nuclear protein lamin B1 in the S10 fractions also indicates that the increase in cytoplasmic La levels is not a result of membrane leakage and is likely a result of H_2_O_2_-induced cytoplasmic shuttling.

### H_2_O_2_ induces La cytoplasmic shuttling

**Fig. 3.**
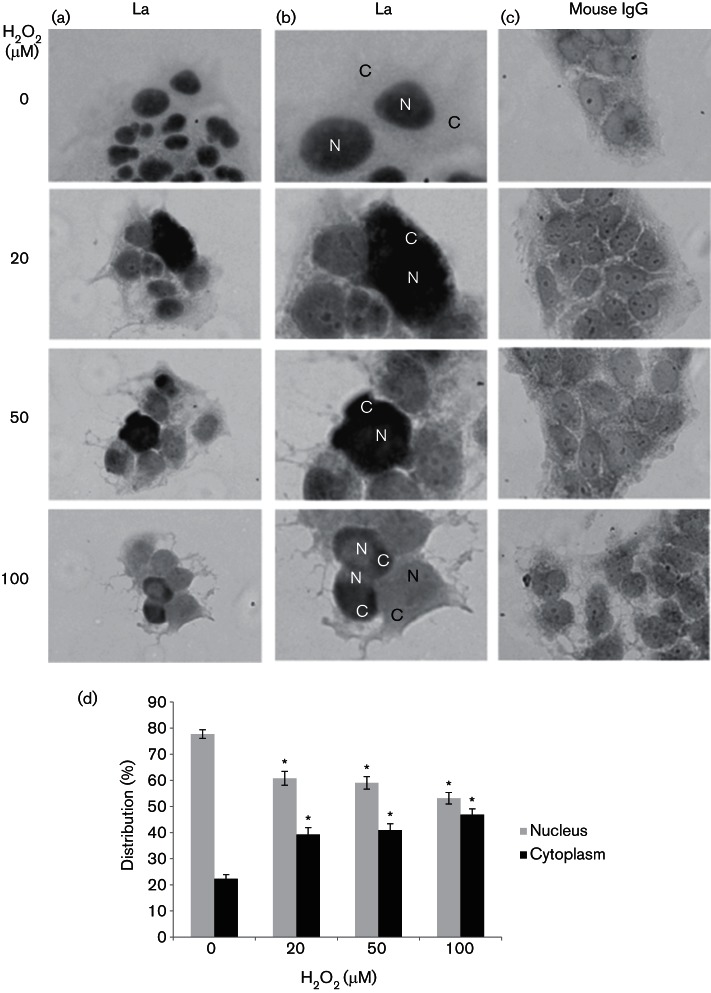
H_2_O_2_ induces La cytoplasmic shuttling. Immunocytochemistry showing the subcellular localizations of the La autoantigen in Huh7 cells treated with doses of H_2_O_2_, as indicated, and in untreated control for 1 h. (a) Representative views of clusters of cells as stained by the anti-La antibody, SW5. (b) Enlarged views of (a) showing details of the staining by the anti-La antibody, SW5. N, Nucleus; C, cytoplasm. (c) Clusters of cells stained with the isotypic control mouse IgG. (d) Percentage distribution of nuclear and cytoplasmic staining of the La protein. The values obtained represent the mean±sem of three independent experiments. Between 140 and 220 cells per treatment were analysed from each of the three repeats. Significance of the difference *P<0.05.

**Fig. 4.**
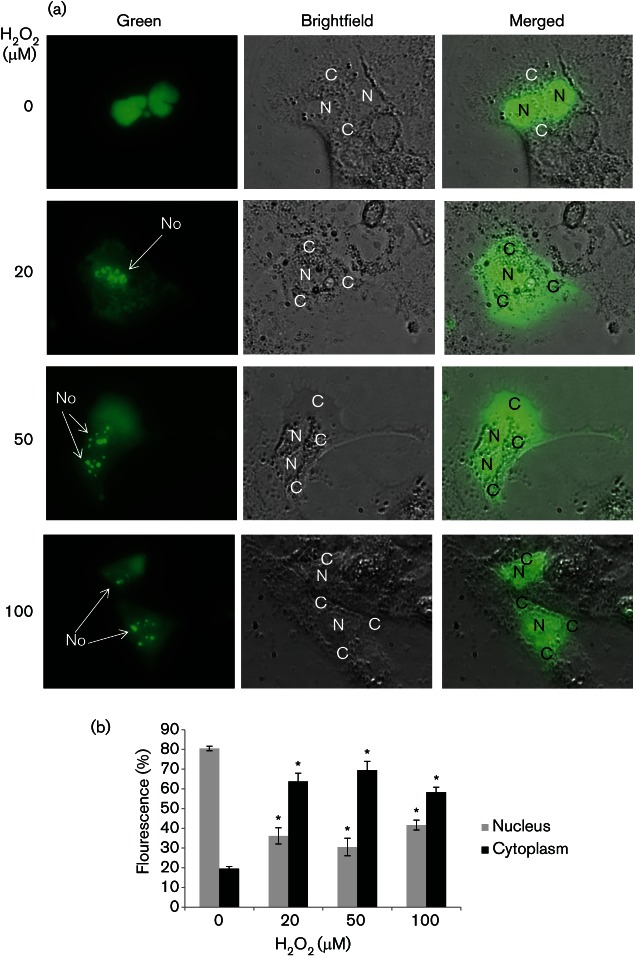
H**_2_**O**_2 _**induces GFP–La cytoplasmic re-localization. (a) Live cell imaging showing subcellular localizations of the GFP–La fusion protein in Huh7 cells treated with doses of H_2_O_2_, as indicated, and in untreated control for 30 min to 2 h. Huh7 cells were transfected with the plasmid expressing GFP–La for 24 h in 24-well plates, then split into 96-well glass-bottomed plate (Nunc) at a density of 19 000 cells per well for 24 h before subjected to treatment with H_2_O_2_. N, Nucleus; C, cytoplasm; No, nucleolus. (b) Percentage distribution of nuclear and cytoplasmic fluorescence of the GFP–La protein. The values obtained represent the mean±sem of three independent experiments. Twenty individual cells per treatment were analysed from each of the three repeats. Significance of the difference *P<0.05.

We then studied whether H_2_O_2_ was able to induce endogenous La re-localization to the cytoplasm, using immunocytochemistry. In untreated cells, the La protein displayed as dark nuclei staining, consistent with the fact that La is an abundant nuclear protein (Fig. 3) (Wolin & Cedervall, 2002). In Huh7 cells treated with 20 µM, 50 µM and 100 µM H_2_O_2_, the nuclear staining was significantly diminished to 61, 59  and 53 %, respectively; compared with that of 78 % in untreated cells. Staining became diffuse and was distributed in both the nucleus and the cytoplasm. Occasionally, dark cytoplasmic staining was observed. Significantly more cytoplasmic staining was detected in 20 µM, 50 µM and 100 µM H_2_O_2_-stressed cells (39 , 41  and 47 %); compared with that of 22 % in non-stressed cells. Altogether, these results suggest that the La protein has re-shuttled to the cytoplasm. Further confirmation was obtained using a GFP-tagged La protein transiently transfected into Huh7 cells (Fig. 4). The GFP–La fusion protein assumed a predominantly nuclear localization in non-stressed cells, resembling that of the endogenous La protein distribution (Fig. 4a). In cells treated with 20 µM, 50 µM and 100 µM of H_2_O_2_, a proportion of the GFP–La had migrated to the cytoplasm, hence, the fluorescence became diffuse. The nucleus was not clearly defined, however, the nucleoli (No) were visible. The nuclear/cytoplasmic (N/C) ratio of the GFP–La protein significantly changed from 80/20 % in non-stressed cells to 36/64 % , 31/69 %  and 42/58 % in 20 µM, 50 µM and 100 µM H_2_O_2_-stressed cells, respectively, confirming cytoplasmic re-localization of the La protein upon exposure to H_2_O_2_ (Fig. 4b).

### Increased cytosolic La corresponds to enhanced La-IRES binding

**Fig. 5.**
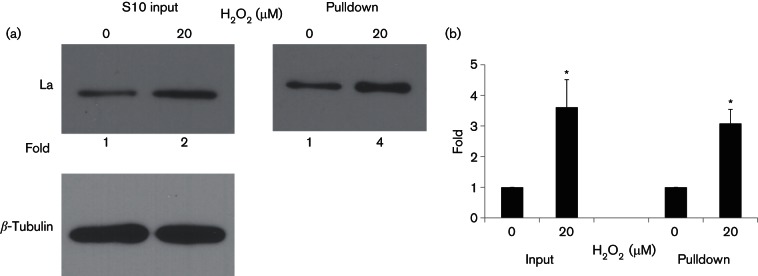
H_2_O_2_ increases binding of La to HCV IRES. Cytoplasmic S10 fractions from untreated and Huh7 cells treated with 20 µM of H_2_O_2_ for 1 h were pulled down by biotinylated HCV IRES RNA, separated on 12 % SDS-PAGE and probed for the La protein using the anti-La antibody 3B9 (1 : 5000) and HRP-conjugated anti-mouse secondary antibody (1 : 5000). The original S10 fractions were run as inputs. *β*-Tubulin (1 : 5000) is the internal loading control. The levels of La were quantified using ImageJ. In the S10 input, the levels of La were normalized against *β*-tubulin and expressed as fold increase relative to the 0 µM H_2_O_2_ control. (a) A representation of the Western blot. (b) The levels of the La protein pulled down by biotinylated HCV IRES RNA. The values obtained represent the mean±sem of three independent experiments. Significance of the difference *P<0.05.

To examine whether the increase in cytoplasmic La level would enhance its binding to IRES RNA to activate HCV IRES-dependent translational activity, we performed an *in vitro* binding assay using the IRES fragment. Biotinylated IRES RNA pulled down three (2.5–4) times significantly more La protein from the cytosolic extract of 20 µM H_2_O_2_-treated cells, compared with that from the untreated control, confirming that the increase in cytoplasmic La level resulted in enhanced binding to the HCV IRES RNA to stimulate IRES-dependent translational activity (Fig. 5).

### La antibody blocks IRES H_2_O_2_ responsiveness *in vitro*

**Fig. 6.**
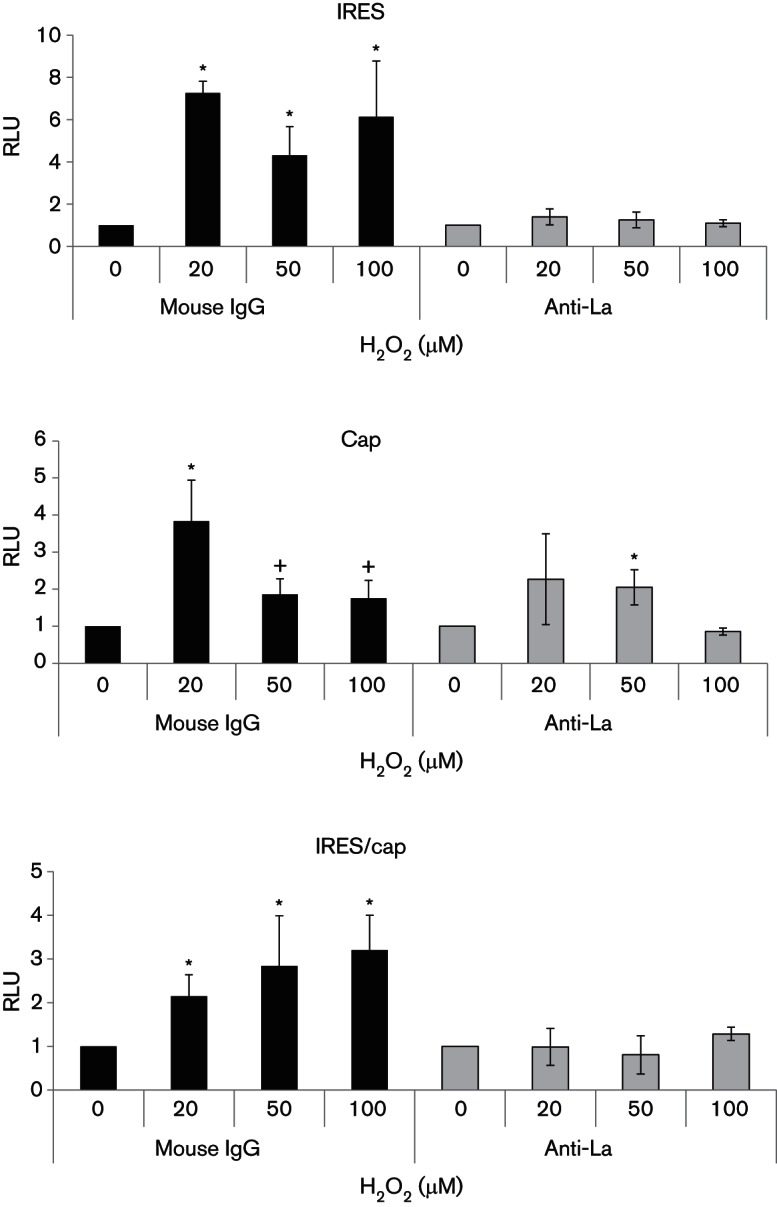
La antibody inhibits H_2_O_2_-activated translation *in vitro*. Cytosolic S10 fractions extracted from Huh7 cells treated with doses of H_2_O_2_, as indicated, for 1 h were incubated with 1 µl of the anti-La antibody SW5, or the control mouse IgG for 1 h on ice before being used to prime *in vitro* translation programmed with the bicistronic pRL1b reporter transcript. The HCV IRES and cap-translational activities were measured by firefly and *Renilla* luciferase activities and expressed relative to their respective 0 µM H_2_O_2_ controls, which are set as 1. The IRES/cap ratio is represented by the ratio of firefly-to-*Renilla* luciferase activities and is expressed relative to their respective 0 µM H_2_O_2_ control, which is set as 1. The values obtained represent the mean±sem of three independent experiments, performed in duplicates. Significance of the difference, *(*P*<0.05), ^+^(*P*<0.1). RLU, Relative luciferase units.

To study whether the increased binding of cytosolic La to HCV IRES RNA was responsible for the enhanced translational activity in H_2_O_2_-treated cells, we examined whether abolishing the effect of La would be able to block IRES responsiveness to H_2_O_2_. To inhibit La function, we added a La-specific antibody, SW5, to the *in vitro* translation reaction. Addition of the anti-La antibody, SW5, but not the isotypic control mouse IgG, to 20 µM, 50 µM and 100 µM of H_2_O_2_-treated cytosolic S10 fractions reduced the IRES-dependent translational activity to the same level as that primed by the 0 µM H_2_O_2_-treated S10 fraction in *in vitro* translation reactions, suggesting that anti-La antibody was able to abolish/reduce the stimulatory effect of H_2_O_2_ on IRES-dependent translation (Fig. 6). Note that the data have been plotted relative to their respective 0 µM controls for ease of comparison. Examination of the absolute values revealed generally lower readings in antibody-treated samples, in agreement with a role of the La protein in HCV IRES-mediated translation (Costa-Mattioli *et al.*, 2004). In contrast, the anti-La antibody only exerted insignificant reduction of the stimulatory effect of H_2_O_2 _on the cap-dependent translational activity in some but not all concentrations of H_2_O_2_, compared with that of the control mouse IgG. Importantly, the addition of the anti-La antibody, but not that of the control mouse IgG, significantly reduced the IRES/cap ratios in the H_2_O_2_-treated samples to the same level as that of the 0 µM H_2_O_2_ sample.

### La-DN mutant blocks IRES responsiveness to H_2_O_2 _*ex vivo*

**Fig. 7.**
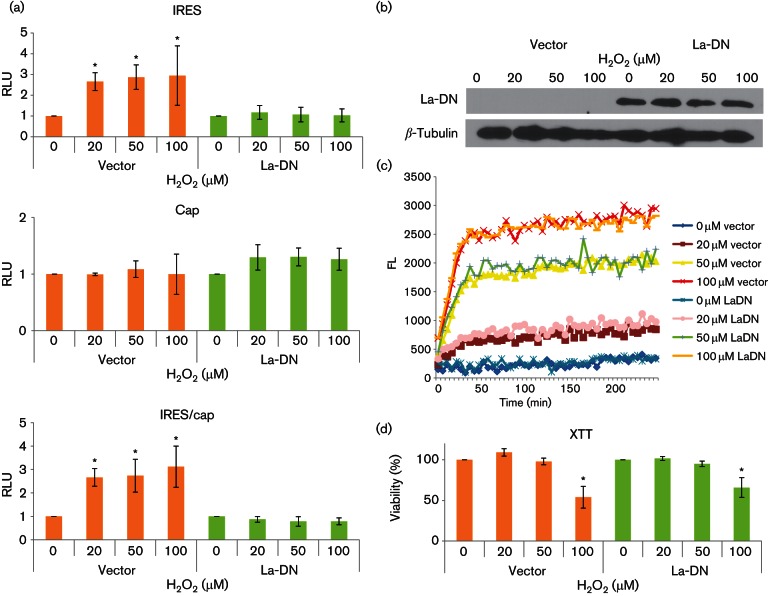
La-DN inhibits H_2_O_2_-activated translation *ex vivo*. (a) Relative translational activities in Huh7 cells transfected with either the dominant-negative La (La-DN) mutant or the control vector for 24 h, split into a 24-well plate at a density of 1.2×10^5^ for 24 h before being transfected with the bicistronic pRL1b RNA transcript for 4 h followed by treatment with the indicated doses of H_2_O_2_ for 4 h. The HCV IRES and cap-translational activities were measured by firefly and *Renilla* luciferase activities, normalized with respect to total protein, and expressed relative to their respective 0 µM H_2_O_2_ controls, which are set as 1. The IRES/cap ratio is represented by the ratio of firefly to *Renilla* luciferase activities and is expressed relative to their respective 0 µM H_2_O_2_ control, which is set as 1. The values obtained represent the mean±sem of three independent experiments, performed in triplicates. RLU, Relative luciferase units. (b) Western blots showing expression of La-DN (detected by the myc-tag antibody clone A-14 Santa Cruz 1 : 1000; HRP-conjugated anti-rabbit antibody, 1 : 1000) at 48 h post-transfection and the internal loading control *β*-tubulin (1 : 5000; HRP-conjugated anti-mouse antibody 1 : 1000). (c) A representation of three independent dichlorofluorescin fluorometric assays, performed in triplicates, showing the kinetics of ROS generation in plasmid-transfected Huh7 cells (1.2×10^5^ per well in a 24-well plate) after treatment with doses of H_2_O_2_, as indicated. FL, Fluorescence units. (d) XTT assay showing viability of plasmid-transfected Huh7 cells (1.2×10^5^ per well in a 24-well plate) after treatment with doses of H_2_O_2_, as indicated, for 24 h. The values obtained represent the mean±sem of three independent experiments, performed in triplicates, and are expressed relative to the respective 0 µM H_2_O_2_ controls, which are set as 100 %. Significance of the difference *P<0.05.

To confirm the role of La in HCV IRES H_2_O_2_ responsiveness *ex vivo*, we made use of a dominant-negative (DN) mutant to block the function of endogenous La. Oligomerization is required for La function and the La 226–348 mutant consisting of the dimerization domain functions as a DN mutant, as has been demonstrated before (Costa-Mattioli *et al.*, 2004; Craig *et al.*, 1997; Holcik & Korneluk, 2000). The La-DN mutant, but not that of the vector control, was able to suppress HCV IRES-dependent translational activation as well as the IRES/cap ratio as a result of H_2_O_2_ treatment (20 µM, 50 µM and 100 µM) in Huh7 cells to the same level as that of the 0 µM H_2_O_2_ sample (Fig. 7a). Note that the data have been plotted relative to their respective 0 µM controls for ease of comparison. Examination of the absolute values revealed generally lower readings in La-DN-transfected samples, in agreement with a role of the La protein in HCV IRES-mediated translation (Costa-Mattioli *et al.*, 2004). There was no significant increase in cap-dependent translation in cells transfected with either La-DN mutant or the vector control, confirming the specific effect of H_2_O_2_ on IRES-mediated translation in an *ex vivo* model. Expression of the La-DN mutant was confirmed by the detection of the myc-tag (Fig. 7b). We also confirmed that expression of the La-DN mutant did not significantly alter cells’ responses to H_2_O_2_ challenge compared with that of the vector control, in terms of generation of intracellular oxidants or viability, excluding the possibility that expression of the La-DN might have an effect on cell stress response (Fig. 7c, d).

### La-DN mutant blocks IRES responsiveness to H_2_O_2_ in a sub-genomic replicon cell line

**Fig. 8.**
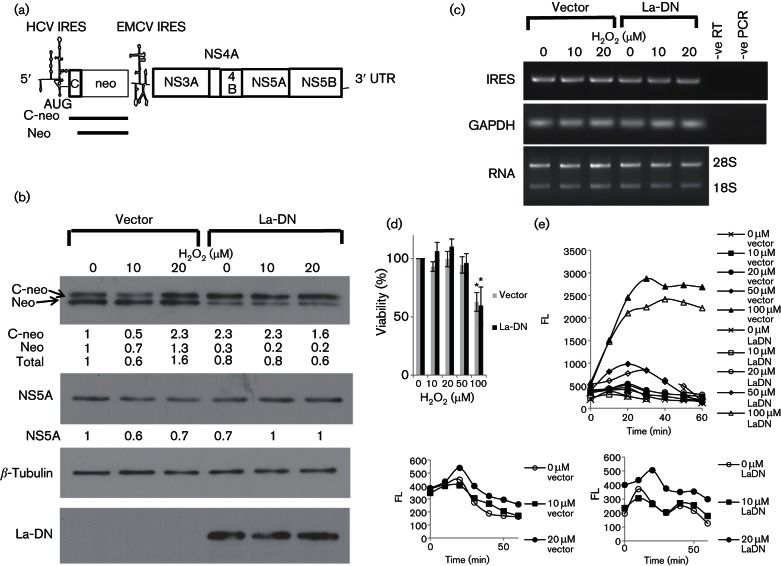
La-DN inhibits H_2_O_2_-activated translation in a sub-genomic replicon cell line. (a) Diagrammatic representation of the sub-genomic replicon used in this study. The sub-genomic replicon consists of a bicistronic transcript in which expression of the core (1–15 aa)-neomycin phosphotransferase (C-neo) fusion protein is under the translational control of the HCV IRES element, whereas expression of the HCV non-structural (NS) 3-NS5B polyprotein is under the translational control of the EMCV IRES element. (b) Western blots showing the levels of the C-neo/neo proteins, NS5A, myc-tag dominant-negative La (La-DN) mutant protein and the internal loading control *β*-tubulin in whole-cell lysates of Huh8 cells transfected with either the empty vector or a vector expressing the La-DN mutant protein and treated with doses of H_2_O_2_, as indicated, for 1 h. The following concentrations were used: (anti-neo antibody clone 4B4D1, Sigma, 1 : 1000; anti-mouse HRP 1 : 500); (NS5A clone 9E10 1 : 50; anti-mouse HRP 1 : 1000); (anti-myc clone A-14, Santa Cruz 1 : 1000; anti-rabbit HRP 1 : 1000); (anti-*β*-tubulin clone TUB2.1, Sigma 1 : 1000; anti-mouse HRP 1 : 1000). The levels of C-neo, neo and NS5A were quantified using ImageJ, normalized against *β*-tubulin and expressed as fold increase relative to the 0 µM H_2_O_2_ vector control. (c) Agarose gel electrophoresis showing the amplified fragments from RT-PCR using primers specific for the HCV IRES element and the internal control glyceraldehyde 3-phosphate dehydrogenase (GAPDH) in Huh8 cells transfected with either the empty vector or a vector expressing the La-DN mutant protein and treated with doses of H_2_O_2_, as indicated, for 1 h. –ve RT, Negative control for the RT reaction; –ve PCR, negative control for the PCR. The bottom panel shows equal quantity of RNA had been used in the RT-PCR. The bands are the 28S and 18S ribosomal RNAs. (d) XTT assay showing viability of Huh8 cells (16 000 per well/96-well plate) after treatment with doses of H_2_O_2_, as indicated, for 24 h. The values obtained represent the mean ± sem of three independent experiments, performed in quadruplicates, and are expressed relative to the untreated control, which is set as 100 %. Significance of the difference *P<0.05. (e) A representation of three independent dichlorofluorescin fluorometric assays, performed in quadruplicates, showing the kinetics of ROS generation in Huh8 cells (16 000 per well/96-well plate) after treatment with doses of H_2_O_2_, as indicated. The bottom part of the graph is enlarged and depicted below to show ROS generation in the lower range of H_2_O_2_. FL, Fluorescence units.

To examine the relevance of the responsiveness of IRES-mediated translation to H_2_O_2_ to the HCV life cycle, we made use of a cell line, Huh8, which harbours an HCV sub-genomic replicon resembling a persistent infection (Blight *et al.*, 2000) (Fig. 8a). The sub-genomic replicon consists of a bicistronic transcript in which expression of the core (1–15 aa)-neomycin phosphotransferase (C-neo) fusion protein is under the translational control of the HCV IRES, whereas expression of the HCV NS3-NS5B is under the translational control of the encephalomyocarditis virus (EMCV) IRES. The effects of H_2_O_2_ on HCV IRES-mediated translation were compared in Huh8 cells transfected with either a plasmid expressing the La-DN protein or the empty vector using Western blotting on whole-cell lysates. We noticed a 26/28 kDa doublet of neo protein (Fig. 8b). The ~2 kDa difference in size would correspond to the estimated 1.6 kDa molecular weight of the N-terminal C peptide MSTNPKPQRKTKGRA. We reckoned that the upper band represents the C-neo fusion protein translated from the authentic start codon of the HCV IRES, whereas the lower band corresponds to neo protein translated from the internal neo start codon. This is possible because the start codon of the neo gene is retained in the C-neo fusion gene and is placed in a favourable Kozak context (Kozak, 1987) (Fig. 8a). Importantly, translation from the HCV IRES was increased by ~twofold in cells exposed to 20 µM H_2_O_2_ over that of the untreated cells in vector-transfected cells, confirming that translation from the HCV IRES is responsive to H_2_O_2_ in the context of viral replication resembling a persistent infection. Moreover, the effect of H_2_O_2_ was specific towards translation from the authentic start codon as it induced a 2.3-fold increase in the C-neo protein as compared with only a 1.3-fold increase in the neo protein (translated from the internal start codon). In contrast to Huh7 cells, which responded to 10 µM of H_2_O_2_ by increasing HCV IRES translational activity from a transiently transfected bicistronic reporter transcript (see Fig. 1a), treatment of vector-transfected Huh8 cells with 10 µM of H_2_O_2_ was not enough to increase the HCV IRES translational activity compared with that of the untreated control, as measured by the C-neo/neo levels (Fig. 8b). It is tempting to speculate that the two cell lines have different thresholds of H_2_O_2_ responsiveness. Indeed, 10 µM of exogenous H_2_O_2_ was sufficient to raise the intracellular oxidant level in Huh7 cells but not that in Huh8 cells (compare Figs 1b and 8e). To exclude the possibility that the increase in C-neo expression in 20 µM H_2_O_2_-treated cells was a result of increased replication of the sub-genomic replicon induced by H_2_O_2_, we analysed the sub-genomic replicon RNA levels using reverse transcription (RT)-PCR and primers specific for the HCV RNA. We showed similar levels of the sub-genomic replicon RNA in cells regardless of H_2_O_2_ treatments, confirming that H_2_O_2_ induced translation from the HCV IRES in the sub-genomic replicon cell line (Fig. 8c). In contrast, translation of the NS5A protein from the EMCV IRES was reduced upon H_2_O_2_ treatments, indicating that translation from the EMCV IRES is inhibited by H_2_O_2_ and responsiveness to H_2_O_2_ is specific to the HCV IRES element and is not a general phenomenon for all viral IRESs.

We then investigated whether La-DN mutant could inhibit HCV IRES H_2_O_2_ responsiveness by using transient transfection of Huh8 with a plasmid expressing the La-DN mutant (Holcik & Korneluk, 2000). We do not know why there was a preferential translation from the internal neo start codon in the vector-transfected cells, but from the authentic C-neo start codon in the La-DN-transfected cells, however, comparison of total C-neo and neo levels in untreated cells showed that expression of La-DN did reduce the total neo level, confirming that the La-DN mutant did exhibit a DN effect on HCV IRES-mediated translation (Fig. 8b). The modest DN effect could be a result of cultivation of Huh8 cells in the presence of G418 (neomycin) and, hence, it was essential to maintain expression of the neomycin-resistant protein, i.e. C-neo/neo. Importantly, we showed that expression of La-DN abolished responsiveness of HCV IRES-mediated translation to 20 µM of H_2_O_2_, confirming that La plays a role in H_2_O_2_-induced translation from the HCV IRES in the sub-genomic replicon. The sub-genomic replicon RNA levels remained similar regardless of transfection or treatments, confirming that the effect of H_2_O_2_ is at the level of translation and not at the level of viral replication (Fig. 8c). We also confirmed that expression of the DN-La mutant did not significantly alter cells’ responses to H_2_O_2_ challenge compared with that of the vector control, in terms of generation of intracellular oxidants or viability, excluding the possibility that expression of the La-DN might have an effect on cell stress response (Fig. 8d, e).

## Discussion

In this study, we showed that translation from the HCV IRES was upregulated by a range of physio/pathological levels of H_2_O_2_ that could be encountered by the virus during its prolonged phase of chronic infection, which has significance to HCV survival in an environment with continuously fluctuating levels of oxidants. This is similar to the human immunodeficiency virus (HIV), which also establishes a chronic infection and is associated with elevated oxidative stress in patients and enhanced IRES-dependent translation upon oxidative stress, suggesting that these viruses can adapt to and utilize oxidative stress to aid in their translation (Gendron *et al.*, 2011; Jack & Chan, 2011; MacCallum *et al.*, 2006; Stehbens, 2004). This could be a novel survival mechanism used by viruses to evade oxidative stress and establish chronicity.

The ability to respond to H_2_O_2_ is specific to certain viruses and is not a general characteristic of all viral IRESs, as we showed that translation from the EMCV IRES was inhibited by H_2_O_2_. The HCV IRES element is more closely related to the picornaviral IRESs than the HIV IRES. It has been speculated that HCV acquired an IRES element from picornavirus in the distant past by recombination (Belsham, 2009; Hellen & de Breyne, 2007). The HCV IRES has been grouped with Type IV picornaviral IRESs to form a class of HCV-like IRESs based on structural and functional similarity (Sweeney *et al.*, 2012). The fact that both HCV and HIV (and some cellular IRESs – see below), but not EMCV IRES-mediated translation is responsive to H_2_O_2,_ leads us to speculate that IRES responsiveness to oxidative stress is a convergent function evolved with persistent infection/stress response. However, the EMCV IRES is a type II picornaviral IRES, which is structurally and functionally distinct from the HCV-like IRES (Belsham, 2009). Interestingly, basal translation from the poliovirus IRES (a type I picornaviral IRES) is regulated by the La protein – the same ITAF that is responsible for H_2_O_2_-inducible translation from the HCV and the cellular nuclear factor erythroid-2-related factor 2 (Nrf2) IRESs (Belsham, 2009; Costa-Mattioli *et al.*, 2004; Li *et al.*, 2010). Therefore, further studies need to be carried out using picornaviral and HCV-like IRESs to see whether responsiveness to oxidative stress is a function preserved in all HCV-like IRESs and some picornaviral IRESs, regardless of whether they establish an acute or chronic infection or it is a function evolved with persistent infection.

Apart from the HCV IRES, other IRESs that have been investigated thus far also exhibit translational upregulation in response to oxidative stress (Daba *et al.*, 2012; Gendron *et al.*, 2011; Giudetti *et al.*, 2013; Li *et al.*, 2010; Yeh *et al.*, 2011; Zhang *et al.*, 2010, 2012). Still, exactly how oxidative stress stimulates IRES-dependent translation is far from clear. Despite being collectively known as IRES, each IRES is unique in terms of sequence, structure, use of eIF and ITAF, mechanism of translation and response to stress (Plank & Kieft, 2012). Thus, it is anticipated that the mechanisms used to respond to oxidative stress would be as diverse as the IRES itself. So far, a positive regulatory mechanism and a derepression mechanism have been proposed for the H_2_O_2_-responsive Nrf2 and HIV IRES-dependent translation, respectively (Gendron *et al.*, 2011; Li *et al.*, 2010). We, and others have shown that the La protein is responsible for translational upregulation from the HCV and Nrf2 IRESs during transient and prolonged oxidative stress (Zhang *et al.*, 2012; and this study). The mechanistic similarity between HCV and Nrf2 IRESs suggests that IRES oxidative stress responsiveness could be an evolutionarily conserved homeostatic/adaptive response. Indeed, translation from the IRESs of Nrf2 and ferritin, both of which are important regulators in restoring redox balance, is stimulated by pro-oxidants (Daba *et al.*, 2012; Li *et al.*, 2010; Zhang *et al.*, 2012). A protective response to oxidative stress was also mediated by Sp1 IRES-dependent translational upregulation in a pathological setting of ischaemic insults (Yeh *et al.*, 2011).

Post-translational modification is commonly used to regulate ITAF function during stress (Brenet *et al.*, 2009; Lewis & Holcik, 2008; Sella *et al.*, 1999; Shiroki *et al.*, 1999). La is an abundant nuclear protein, and nuclear–cytoplasm shuttling represents a rapid means of regulation of its function (Lewis & Holcik, 2008). Cytoplasmic export of La is accelerated during poliovirus infection, apoptosis and immune activation, by the cleavage of the C-terminal nuclear localization signal by viral serine protease, caspase and granzymes (Huang *et al.*, 2007; Romero *et al.*, 2009; Shiroki *et al.*, 1999). For one of the doses of H_2_O_2_ we used (100 µM) to induce oxidative stress, which also caused apoptosis, however, our Western blots did not reveal any noticeable truncation of the La protein within the time frame of our study, suggesting that oxidative stress-induced apoptotic cleavage of the La protein is not responsible for La shuttling to stimulate HCV IRES-dependent translation in Huh7 cells in our case. Indeed, it has been found that cytoplasmic shuttling of cleaved La during apoptosis actually inhibits, rather than stimulates, HCV IRES-dependent translation (Romero *et al.*, 2009). In another study, a 10 min transient H_2_O_2_ treatment of an apoptotic dose of H_2_O_2_ (100 µM) on HeLa cells was also sufficient to induce La shuttling without truncation (Zhang *et al.*, 2012). This is further supported by the demonstration, in our study, that La shuttling also occurred in non-apoptotic doses of H_2_O_2_, 10 µM and 20 µM, in Huh7 cells (Figs 2, 3 and 4). Thus, a mechanism involving transportation of full-length La is highlighted. La is a phosphoprotein and its shuttling between nucleus and cytoplasm is controlled by nuclear localization signal and nuclear export signal (Bayfield *et al.*, 2010). It is possible that de/phosphorylation shuttles La upon stress by altering the nuclear retention element structure to unmask the nuclear export signal (Intine *et al.*, 2003). Dephosphorylation of La has been demonstrated in a number of cell types in response to diverse apoptotic signals (Rutjes *et al.*, 1999). During tumour growth-factor-beta stimulation of epithelial cells and epithelial to mesenchymal transition, platelet-derived growth factor (PDGF) mediated La cytoplasmic shuttling via the MARK/ERK signalling to stimulate translation from the laminin B1 IRES, suggesting the involvement of a phosphorylation event (Petz *et al.*, 2012). Indeed, it has been shown that PDGF-stimulated Akt-phosphorylation of La mediated its nuclear export to regulate a subset of mRNA translation (Brenet *et al.*, 2009). However, a transient stress with 100 µM of H_2_O_2_ did not result in phosphorylation of La in HeLa cells, which might account for its cytoplasmic relocation to stimulate translation from the Nrf2 IRES (Zhang *et al.*, 2012). Altogether, they imply diverse mechanisms used to export nuclear La upon different stress signals and/or in different cell types.

Intriguingly, we detected a neo protein translated from an internal AUG start codon in addition to the C-neo fusion protein translated from the authentic HCV IRES AUG start codon when we used a sub-genomic replicon cell line resembling a persistent infection to study the response of the HCV IRES-mediated translation to H_2_O_2_ (Fig. 8). It is well known that translation from the HCV IRES can utilize alternative ORFs and internal AUG and non-AUG start codons, reflecting translational flexibility (Boumlic *et al.*, 2011).

On one hand, HCV replication is modulated by oxidative stress (Choi *et al.*, 2004; McCartney *et al.*, 2008); on the other hand, La also plays a role in viral replication (Kumar *et al.*, 2013). However, we still do not yet know whether oxidative stress exerts its effect on viral replication via La. It will be interesting to study, in the future, how oxidative stress affects the dynamics of translation, replication and its role in translation–replication switch.

It is generally believed that H_2_O_2_ inhibits cap-dependent translation (Grant, 2011). Some studies on H_2_O_2_- responsive IRES-dependent translation also detected downregulation of cap-dependent translation (Gendron *et al.*, 2011; Giudetti *et al.*, 2013). In contrast, we, and others have previously detected simultaneous upregulation of cap- and IRES-dependent translation (with increased IRES/cap ratio), suggesting that cap-dependent translation could be upregulated by H_2_O_2_ (Li *et al.*, 2010; MacCallum *et al.*, 2006). In this study, we detected an inconsistent response of cap-dependent translation to H_2_O_2_. It is well known that susceptibility of cells to H_2_O_2_ fluctuates (Dickinson & Chang, 2011; Veal *et al.*, 2007). A major determining factor is the balance between endogenous pro- and anti-oxidant levels/species and the redox buffering capacity, which is, in turn, very much affected by the passage number (age, metabolism, speed and ability to adapt) of the cells. Therefore, the response of cap-dependent translation to H_2_O_2_ could be more varied than previously thought (Davies, 1999; Grant, 2011).

In conclusion, HCV adapts to oxidative stress in the host cell by increasing translation from its IRES as a result of oxidative stress-induced cytoplasmic shuttling of its main ITAF, La. Further work is required to delineate the H_2_O_2_-responsive element in the HCV IRES and to decipher the mechanism by which full-length La re-shuttles to the cytoplasm during oxidative stress.

## Methods

### Cell culture.

Huh7 and Huh8 cells were maintained in Dulbecco’s modified Eagle’s medium, 10 % FCS, 100 µg penicillin ml^−1^, 100 µg streptomycin ml^−1^, 4 mM glutamate and 1× non-essential amino acids. Huh8 cultures were supplemented with 100 mg ml^−1 ^G418 (Melford). To induce oxidative stress, H_2_O_2_ (30 % w/w solution, Sigma) was added exogenously.

### XTT viability assay.

XTT assay was performed according to the manufacturer’s instruction (Cell Proliferation Kit II, Roche). Cells seeded in 24-well/96-well plates were treated with H_2_O_2_ for 24 h. XTT was added and readings were taken at 450 nm using a 650 nm reference filter (Bio-Tek Synergy HT).

### Reactive oxygen species (ROS) measurement.

The generation of intracellular ROS was measured using the probe 2′,7′-dichlorofluorescin diacetate (DCFH-DA, Sigma) and a fluorimeter (Berthold Twinkle), as described previously (MacCallum *et al.*, 2006).

### Plasmids.

The bicistronic construct, pRL1b, encoding an upstream *Renilla* luciferase gene under the control of the T7 promoter, and a downstream firefly luciferase gene under the control of the HCV IRES has been previously described (Collier *et al.*, 1998). To generate the sense IRES fragment used in affinity pulldown, the *Bam*HI fragment containing the HCV IRES was excised from the plasmid pRL1b and subcloned into pcDNA3.1 (Invitrogen) to create the plasmid pIRES. The orientation of the insert has been confirmed by restriction digestion. The green fluorescent protein (*gfp*)*-La* plasmid was kindly provided by Junji Sagara (Ayukawa *et al.*, 2000). The plasmid encoding a dominant negative mutant of the La protein (La-DN) and its empty vector were kindly provided by Martin Holcik (Holcik & Korneluk, 2000).

### Transfection.

Transfection with DNA plasmids was performed using Fugene HD (Promega) according to the manufacturer’s instructions. Transfection with RNA transcripts was performed using Trans IT-mRNA transfection kit (Mirus), according to the manufacturer’s instructions.

### Extraction of total lysates, cytoplasmic S10 and nuclear fractions.

Cells were trypsinized and washed with ice-cold PBS four times. A fraction of the pelleted cells was removed for total protein extraction in RIPA buffer (50 mM Tris pH 8, 150 mM NaCl, 1 % NP40, 0.5 % Na deoxycholate, 0.1 % SDS) or directly into SDS-PAGE loading buffer (62.5 mM Tris pH 6.8, 2 % SDS, 5 % β-mercaptoethanol, 10 % glycerol, 0.002 % bromophenol blue) for 10 min at 100 °C followed by clarification for 10 min at 14 000 rpm, 4 °C. To obtain S10, the remaining cell pellet was lysed in ice-cold hypotonic buffer (10 mM K-Hepes pH 7.5, 10 mM KOAc, 1.5 mM MgOAc, 2.5 mM DTT) for 10 min. The lysate was centrifuged at 1000 ***g*** for 5 min to pellet the nuclei and cell debris. The supernatant was then centrifuged at 10 000 ***g*** for 20 min to obtain the cytoplasmic S10 fraction. To obtain the nuclear fraction the pellet was washed twice with hypotonic buffer and then resuspended in RIPA buffer. Nuclear fraction was extracted by rotation for 30 min, 4 °C; followed by incubation at 100 °C for 10 min and then clarification for 20 min at 14 000 rpm, 4 °C. The protein concentrations of the S10 fractions were determined using the Bio-Rad Bradford protein assay according to the manufacturer’s instructions. The protein concentrations of the total lysates and nuclear fractions were determined using the BCA kit (Sigma) or the RC-DC kit (Bio-Rad) according to the manufacturers’ instructions.

### *In vitro* transcription.

The bicistronic reporter RNA template used in *in vitro* translation reactions was generated by *in vitro* transcription from the T7 promoter of pRL1b using the Ambion’s mMESSAGE mMACHINE transcription kit according to the manufacturer’s instructions. Biotinylated RNA used in *in vitro* affinity pulldown reactions was synthesized from linearized pIRES plasmid DNA using the Ambion’s T7 Megascript transcription kit according to the manufacturer’s instructions, with the addition of 7.5 mM ATP, 7.5 mM CTP, 7.5 mM GTP, 5.6 mM UTP and 1.9 mM biotin-16-uridine-5′-triphosphate (Roche).

### *In vitro* translation.

*In vitro* translation was carried out at 37 °C for 1 h using equal quantities of the S10 extracts and 0.4 µg of the RNA template in 10 µl of reaction mixture containing 6.4 mM creatine phosphate, 0.08 µg creatine phosphokinase, 1.6 mM DTT, 14.2 mM Hepes-KOH pH 7.2, 0.2 mM spermidine, 170 mM KOAc, 1.5 mM MgOAc, 1 mM ATP, 20 µM GTP, 20 µM complete amino acids mix (Promega) and 0.2 µl of RNase inhibitor (New England Biolabs). An aliquot of the reaction (5–10 µl) was used to measure the firefly and *Renilla* luciferase activities.

### Dual luciferase assay.

The activities of firefly and *Renilla* luciferases were measured in relative light units over 10 s with a luminometer (Berthold Lumat LB9507), using the Dual-Luciferase Reporter Assay System (Promega) according to the manufacturer’s instructions.

### RNA affinity pulldown.

Biotinylated RNA (10 µg) was mixed with 100 µg of S10 fraction in 500 µl binding buffer containing 10 mM Tris pH 7.4, 150 mM KCl, 1.5 mM MgCl_2_, 0.5 mM DTT, 1 : 100 protease inhibitor cocktail (Sigma), 0.05 % NP40, 30 µg yeast tRNA (Sigma), 1 µl RNase inhibitor (New England Biolabs) for 30 min at room temperature followed by 2 h at 4 °C with slow rotation. To pulldown biotinylated RNA, 100 µl (1 : 1 slurry) of pre-washed streptavidin agarose beads (CL-4B, Sigma) were added to each reaction, and incubation continued at 4 °C for 2 h with slow rotation. The beads were then washed five times with binding buffer and the pellets resuspended in 20 µl of SDS-PAGE loading buffer for electrophoretic separation on 12 % SDS-PAGE gel and Western blotting.

### Western blotting. 

Western blotting was performed as previously described (Chan & Egan, 2005, 2009). Proteins separated on 12 % SDS-PAGE gel were incubated with primary antibodies, followed by HRP-conjugated secondary antibodies (Cell Signaling) in 5 % semi-skimmed milk (Marvel)/0.1 % Tween 20/TBS. Immunocomplexes were detected with the Uptilight HS HRP chemiluminescence system (Uptima).

### Immunocytochemistry.

Cells grown on chamber slides (Falcon) were fixed in ice-cold methanol for 1 h. After blocking for 1 h in 1 % horse serum/2 % BSA/PBS, cells were incubated with anti-La antibody SW5 (1 : 10) or isotypic control, and then HRP-conjugated secondary antibody (1 : 50; Cell Signaling) for 1 h each in blocking buffer. Colour was developed using SIGMA *FAST*^™^ DAB (3,3′-diaminobenzidine tetrahydrochloride).

### Microscopy.

Immunocytochemistry images were collected on a Zeiss Axioskop upright microscope using a 40×/0.95 Plan Apochromat objective and captured using an Axiocam colour CCD camera through Zeiss Axiovision software. GFP images were collected on an Zeiss Axiovert inverted microscope using a 60×/1.25 Plan Neofluar objective and captured using a Sony CCD video camera through Zeiss Axiovision software. Images were then processed and analysed using ImageJ (http://rsb.info.nih.gov/ij).

### RNA extraction.

RNA was extracted from cultured cells using RNA-Bee (AMSBIO) according to the manufacturer’s instruction. RNA was quantified using a Nanodrop 1000.

### Reverse transcription (RT)-PCR.

RNA (1 µg) was incubated with 1.5 µl of 20 µM antisense (AS) primer at 70 °C for 5 min and then added to a total volume of 20 µl of RT reaction consisting of 50 mM Tris pH 8.3, 75 mM KCl, 3 mM MgCl_2_, 10 mM DTT, 0.24 mM dNTP, 3 µl DMSO, 0.1 µl RNase inhibitor (New England BioLabs) and 1 µl of Moloney murine leukemia virus reverse transcriptase (Promega) at 42 °C for 1 h. An aliquot (1 µl) of RT reaction was used in a total volume of 20 µl of PCR consisting of 20 mM Tris pH 8.8, 10 mM (NH4)_2_SO4, 10 mM KCl, 2 mM MgSO4, 0.1 % Triton-X-100, 0.2 mM dNTP, 1.25 µM each of sense and AS primers and 0.1 µl *Taq* polymerase (New England BioLabs). PCR was carried out with a 2 min denaturation and 25 cycles of (94 °C/25 s; 55 °C/35 s; 68 °C/1 min) followed by a 7 min extension at 68 °C. The sense and AS primers used to amplify the IRES fragment were 5′-caccagatctcactcccctgtgaggaacta-3′ and 5′-tgcccagtcatagccgaatag-3′. The sense and AS primers used to amplify the glyceraldehyde 3-phosphate dehydrogenase (GAPDH) fragment were 5′-cctgttcgacagtcagccg-3′ and 5′-cgaccaaatccgttgactcc-3′.

### Statistics.

Statistical analyses were performed using ANOVA. A *P* value <0.05 was considered significant.
